# Disruption of a Quorum Sensing mechanism triggers tumorigenesis: a simple discrete model corroborated by experiments in mammary cancer stem cells

**DOI:** 10.1186/1745-6150-5-20

**Published:** 2010-04-20

**Authors:** Zvia Agur, Yuri Kogan, Liora Levi, Hannah Harrison, Rebecca Lamb, Oleg U Kirnasovsky, Robert B Clarke

**Affiliations:** 1 Institute for Medical BioMathematics, 10 Hate'ena St, POB 282, 60991, Bene Ataroth, Israel; 2 Breast Biology Group, Division of Cancer Studies, Faculty of Medicine and Human Sciences, University of Manchester, Christie Hospital NHS Trust, Manchester, UK

## Abstract

**Background:**

The balance between self-renewal and differentiation of stem cells is expected to be tightly controlled in order to maintain tissue homeostasis throughout life, also in the face of environmental hazards. Theory, predicting that homeostasis is maintained by a negative feedback on stem cell proliferation, implies a Quorum Sensing mechanism in higher vertebrates.

**Results:**

Application of this theory to a cellular automata model of stem cell development in disrupted environments shows a sharply dichotomous growth dynamics: maturation within 50-400 cell cycles, or immortalization. This dichotomy is mainly driven by intercellular communication, low intensity of which causes perpetual proliferation. Another driving force is the cells' kinetic parameters. Reduced tissue life span of differentiated cells results in uncontrolled proliferation. Model's analysis, showing that under the Quorum Sensing control, stem cell fraction within a steady state population is fixed, is corroborated by experiments in breast carcinoma cells. Experimental results show that the plating densities of CD44+ cells and of CD44+/24lo/ESA+ cells do not affect stem cell fraction near confluence.

**Conclusions:**

This study suggests that stem cell immortalization may be triggered by reduced intercellular communication, rather than exclusively result from somatic evolution, and implies that stem cell proliferation can be attenuated by signal manipulation, or enhanced by cytotoxics targeted to differentiated cells. *In vivo *verification and identification of the Quorum Sensing mediating molecules will pave the way to a higher level control of stem cell proliferation in cancer and in tissue engineering.

**Reviewers:**

This article was reviewed by Glenn Webb and Marek Kimmel.

## Background

In normal tissues, the balance between the unlimited self-renewal capacity of stems cells (SCs), and their ability to continuously supply the required number of end cells, is regulated by numerous environmental signals, acting through paracrine or autocrine pathways [1-3]. This balance is expected to be tightly controlled in order to maintain tissue homeostasis throughout life, also in the face of environmental hazards.

Deciphering the cues that enhance SC proliferation under environmental disturbances, and the cues that attenuate accelerated proliferation when normal conditions resume, may shed light on the origin of cancer and may suggest new methods for its control. The power of the mathematical approach to this problem lies in its simplification. The precision, universality and objectivity of the system's analysis are reinforced by the mathematical model's unique ability to overlook less critical processes.

The simplest model of self-renewable tissues, which succeeds in capturing tissue homeostasis and the proliferation/differentiation properties of individual SCs was presented in [4]. Mathematical analysis of this simple discrete model rigorously proves that long-term tissue homeostasis is guaranteed by a negative feedback control of cell density on SC proliferation. Put simply, SC proliferation can take place as long as the number of SCs in the micro-environment is smaller than a given threshold and sufficient space for replication exists. Transition of a SC from proliferation to differentiation occurs when the number of SCs in its micro-environment is above this specified threshold. A prerequisite for satisfying this condition is a form of cellular *QS *mechanism by which a SC "counts" the number of SCs in its proximity. Such a mechanism characterizes bacteria, e.g., *Vibrio harveyi *and *Vibrio cholera *[5], and is implicated in Burkitt lymphoma [6].

Further mathematical analysis proves that no model, simpler than the *QS *model, can retrieve tissue homeostasis. Tissue homeostasis is defined here as the ability of a few stem cells to repopulate the tissue after severe perturbations, maintenance of a fixed cellular tissue composition and an [almost-] steady-state production of end cells [7]. A recent, more complex, SC model, focusing on quiescence and variability in SC activity, suggests that these properties may be accounted for by natural selection acting on the decisions of stem cells in response to the signals from other SCs in the local micro-environment, and from the more differentiated cells in the rest of the organism [8].

Theoretically, the Quorum Sensing (QS) mechanism may be disrupted by any condition which prevents a faithful *"count" *of SC neighbors. This can be either due to reduced sensitivity of the SC itself, e.g., shortage of adequate receptors for environmental signals, or due to reduced *"clarity" *in the environment, concealing extracellular signals from the SC. The result in both cases is weakened ability to sense the "true" number of SCs in the micro-environment and, as a consequence, incessant proliferation and elusion of normal homeostatic tissue control. These two properties can be integrated into one parameter, the magnitude of intercellular communication sensed by a SC, which is expected to be the critical determinant of the tissue's steady-state production of end cells.

We hypothesized that cancer initiation is driven by disruption of the *QS *mechanism, either by genetic mutations, complying with the current notion of cancer evolution, or purely by the environment, genetic mutations being only a side-effect of excessive proliferation.

The latter suggestion may be supported by evidence about the emergence of intestinal cancer in migrating bone marrow-derived SCs (BMDCs). BMDCs often home to sites of tissue injury and inflammation where they undergo increased proliferation and differentiation [9-12]. It has been suggested that chronic *Helicobacter pylori *infections attract BMDCs to the intestine, where they populate the gastric mucosa and over time contribute to metaplasia, dysplasia, and cancer [13], due to inappropriate retention of BM growth programs in the recruited SCs [14]. Spencer *et al*. [15] propose that tumorigenesis is caused by a different mechanism, namely, by mutant cells within the tissue, out-competing genuine SCs. We will show here that genetic mutation, or any other form of inter-cell variability, is not a prerequisite for tumorigenesis. In the present work we focused on the study of tumorigenesis in normal cells that encounter a new environment. To this end we modified the model in [4], by considering normal SCs homing to a new environment, e.g., BMDC recruitment to a damaged tissue. The new model, having a geometrical tissue structure, was simulated to examine how the proliferation-differentiation balance of the SCs, and the homeostatic properties of the tissue, may be affected by the intensity of intercellular communication and by mutations affecting SCs' kinetic parameters.

To verify our model, we carried out *in vitro *experiments in a growing population of breast cancer cells. Our goal was to test the *QS *theory by examining whether the proportion of SCs in a steady state cell population does not depend on their initial proportion, as the *QS *theory predicts. Having obtained a fixed proportion of SCs in an admixed cell breast carcinomal cell population at confluence, we concluded that the *QS *theory was confirmed by the laboratory experiments.

## Results and Discussion

### Model Analysis

**Figure 1:**
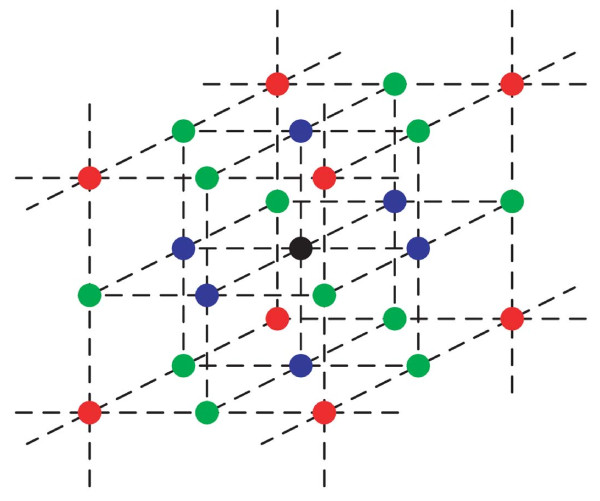
**Geometric structure of the modelled tissue**. The intensity of signal reaching the considered SC (black circle) from SCs in the first, most proximal, layer (blue), second layer (green) or third layer (red) is determined by the respective signal intensity coefficients *K*_1_, *K*_2 _or *K*_3_. Note that the drawn cell sizes are insignificant and that nodes may be occupied by differentiated cells.

Figure 1 schematically represents our tissue model, where cells are embedded in a cellular matrix, represented by a graph. Unlike the previous model [4], the graph in the present model has a distinct geometrical structure, defined by the coordinates of sites, which can be occupied by cells. A micro-environment is characterized by the magnitude of intercellular communication, *K*_*i*_, which stands for the intensity of signals reaching a SC from SCs at a distance of *i*^th ^level. The parameter *K*_*i *_accounts for both properties of the medium and of the molecular signal itself, such as diffusibility and half-life. Similarly to bacterial *QS *[16], a model SC "evaluates" the number of other SCs within its micro-environment by their overall signal intensity, and proliferates as long as signal intensity is sensed as being below a given, fixed, threshold [2,17,18] and there is sufficient space in its vicinity. Transition to differentiation occurs when signal intensity is sensed as being above threshold [4,6]. We denote the SC cycle duration by T, and the obligatory differentiation time by Θ. As our focus in this work is on fate decision of SC, we reduce all differentiated cells, originating from one SC, into one group, denoting it by *DCs*, and its tissue life span by Φ.

Model analysis shows [7] that a quasi-stability of cell numbers within their natural environment exists for the kinetic parameters of the model obeying(1)

This result appears biologically realistic, as the condition in (1) requires that residence time of a differentiated cell line would be larger than SC cycle time; SC cycle time does not include periods of quiescence. Delimiting our investigation to SCs originating in homeostatic tissues alone, we assumed the initial kinetic parameter combinations to obey (1).

In order to simplify our model we ignored competition with resident tissue cells. In this way we mimicked BMDC homing to areas of body injury, where competition with resident SCs can be neglected and the environment is expected to be inflamed, as is the gastric mucosa in gastric ulcer. We performed ~10^4 ^simulations for each environment, examining all possible kinetic properties of the SCs and their progeny. We did so by randomly selecting all the kinetic parameters, discarding kinetic cellular parameter triplets that did not fulfill the inequalities (1). Simulations were carried out for sufficiently long time to ensure full monitoring of all meaningful tissue growth patterns (3·10^3 ^time steps). This period is comparable to 300-3000 days, depending on the SC's average cycle duration.

**Figure 2:**
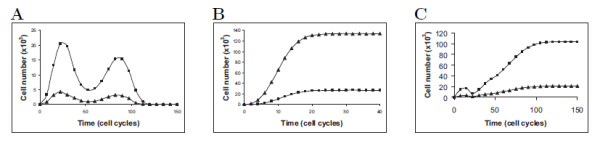
**Types of SC development**. SC development in disrupted environment shows two alternative types of behavior: transient SC proliferation (triangles), production of DCs (squares) and disappearance **(A)**, or rapidly achieved proliferative SC state **(B)**; a case of large DC production and maintenance of a small SC population is rarely achieved **(C)**.

First, we simulated the model under various values of *K*_*i*_, and many cell kinetic parameters, obeying (1). The resulting dynamics show two types of behavior: all the proliferating SCs differentiate and mature within a period of 50-400 cell cycles (Figure 2A), or else, SCs extensively proliferate *"ad infinitum"*, i.e. throughout simulations, switch to differentiation occurring in only a small fraction of them (Figure 2B). These results are interpreted as suggesting that SCs which have evolved in the context of a given environment are very sensitive to alterations in micro-environmental signal intensity, causing them either to fully differentiate within a short time period, or to uncontrollably proliferate, a process which is conducive to cancer [10,13,19]. A third process, maintaining a small SC pool and a large number of DCs, was rarely achieved (Figure 2C).

**Figure 3:**
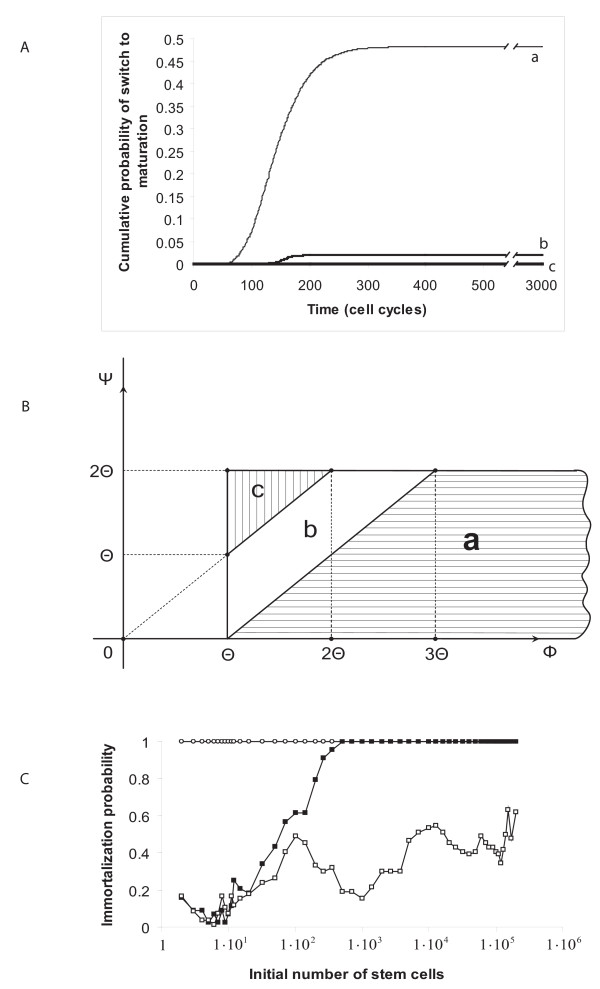
**Factors affecting SC fate**. **(A) **Cells with various kinetic parameters were simulated under different levels of intercellular communication. Three different effects are depicted at steady state. (a) Under a relatively high intercellular communication, *K*_1 _= *K*_2 _= *K*_3 _= 6, *K*_4 _= ... = 0, in about half of the cases all SCs transiently replicate and differentiate into end cells, and in about half of the cases SCs uncontrollably proliferate; (b) Under a lower intercellular communication, *K*_1 _= 6, *K*_2 _= ... = 0, most of the cases evolve to SC immortalization. (c) An even lower intercellular communication, *K*_1 _= *K*_2 _= *K*_3 _= 1, *K*_4 _= ... = 0, or *K*_1 _= 1, *K*_2 _= ... = 0, preserves all SCs in the proliferative state (note two overlapping curves). **(B) **Effect of cell kinetic parameters in environments of high intercellular intensity. Transient SC proliferation and transition to differentiation is dominant when F, Ψ, T obey condition (a) in Table 1. Intermediate probability of full differentiation is obtained under case (b) in Table 1. Immortalization is always obtained under case (c) in Table 1. **(C) **Proportion of immortalized SCs as a function of initial SC density. The parameters Φ, Ψ, Θ obey case (a) in Table I (open squares), case (b) (filled squares), case (c)(circles).

Figure 3A shows the dynamic changes in the fraction of SCs as a function of the level of inter-cellular communication, simulated for an exhaustive variation of tissue parameters. One notices here that in about half of all simulated cases, a relatively intense intercellular communication eventually produces a switch of all SCs in the tissue from a proliferative state to maturation (Figure 3A, a). The probability of a SC to switch to maturation is much lower when signalling intensity in the micro-environment is reduced (Figure 3A, b), whereas in micro-environments having a significantly weak intercellular communication, an uncontrolled proliferation is the only possible outcome (Figure 3A, c).

Our mathematical analysis, summarized in Table 1 and in Figure 3B, suggests that in environments of intense intercellular communication, mutations that affect the cell kinetic parameters, that is, the relations between the periods Φ, Ψ, Θ, will influence SC propensity to uncontrollably proliferate. For all tissues with a given differentiation time, Ψ, and a given life span of DC, Φ, this propensity decreases with increasing SC cycle time, Φ (conditions a, b). Moreover, Table 1(c) and Figure 3B suggest that significantly shortening DC life span, e.g., by differentially eliminating them, may give rise to intensive SC proliferation.

**Table 1:** The expected fates of recruited SCs under intense intercellular communication as depending on the recruited SC kinetic parameters

Class	Transition to maturation	Characteristic parameters	Formal condition
a	Dominant	large DC life span	Ψ ≤ Φ -Θ

b	depends on recruited SC number	intermediate DC life span	Φ - Θ < Ψ ≤ Φ + 1

c	none	DC life span shorter than differentiation time	Ψ > Φ + 1

The initial size of SC population also plays a certain role in affecting tissue structure. Our simulation results may seem counter-intuitive in suggesting that when the initial numbers of SCs that encounter a new environment is large, proliferation may become unlimited (Figure 3C). One can infer, then, that under normal biological circumstances this event is rare, due to the relatively low frequency of SCs in any tissue. This possibility may carry important implications for SC therapy, currently tested for repairing bone, curing cardiovascular diseases and targeting major brain disorders [20-22]. Our results suggest that for preventing excessive SC proliferation in engineered tissues, the initial number of artificially implanted SCs should be limited.

### Experimental Results

The aim of our experiments was to test the *QS *theory underlying the present work. The theory would be refuted if experiments prove that no factors acting specifically on SCs, other than their intrinsic replication rate, determine their fraction in the population at steady state. Under this assumption, the SC fraction at steady state is expected to be correlated with their initial proportion in the population. In contrast, if the *QS *theory is valid, and a threshold number of SC neighbors exists, above which SCs stop replicating, then SC fraction in the overall population at steady state must be fixed, as the mathematical analysis of our model proves [7].

To study this question we relied on the use of CD44, a cell surface adhesion protein whose expression has been reported to allow selective enrichment of breast tumor-initiating cells. Experiments show that the CD44+/CD24 lo/ESA+/cells implanted into NOD/SCID mice form tumors from as few as 200 cells. In contrast, cells with a more differentiated cell surface phenotype (i.e., CD44-/CD24 hi/ESA-) do not form tumors when many thousands of cells are implanted [23].

We isolated SCs, or "stem-like cells," from a breast cancer cell line, using the above markers. The sorted cells were plated with different proportions of either CD44+ or CD44+/24lo/ESA+ cells admixed with the remaining cell populations, and the proportion of SCs was evaluated after 2, 4 or 7 days, when the culture was confluent. This was done both by counting the mammosphere forming units (MFU) and by calculating the proportion of cells bearing the SC marker CD44.

**Figure 4:**
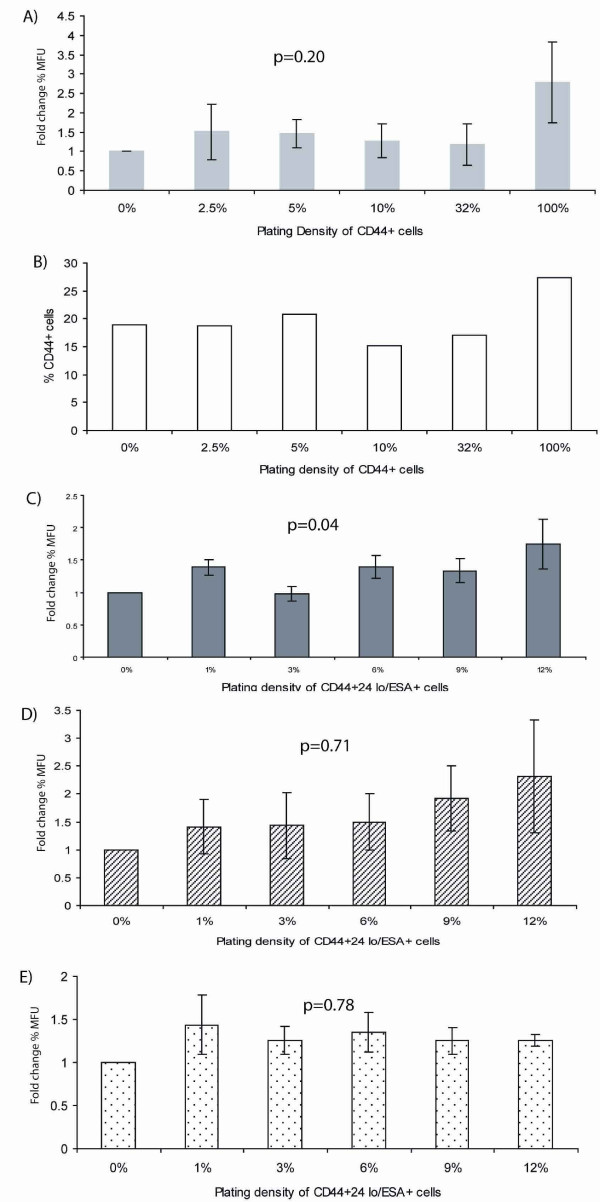
**Experimental results**. **(A) **MCF7 cells were sorted to isolate CD44+ cells and seeded at different proportions (0, 2.5, 5, 10, 32 and 100%) and mixed with the remaining cell population. Cells were cultured in monolayer for 7 days and trypsinised to single cells which were cultured in non-adherent suspension for 7 days. The percentage of mammosphere formation units (%MFU) was counted to determine whether the population achieved a steady state (light grey bars). **(B) **The percentage of cells expressing CD44 after 7 days culture in monolayer was examined using flow cytometry analysis (open bars) **(C) **MCF7 cells were sorted to isolate CD44+/24lo/ESA+ "stem-like" cells and seeded at different proportions (0, 1, 3, 6, 9 and 12%) admixed with the total cell population. Cells were cultured for 2 days (dark grey bars), **(D) **4 days (diagonal striped bars) or **(E) **7 days (dotted bars). Cells were trypsinised to single cells which were cultured in non-adherent suspension for 7 days. The percentage mammosphere formation units (%MFU) was counted. Data was analyzed using analysis of variance (ANOVA) and P values ≤ 0.05 were assumed to be significant.

In the first experiment we used the cell surface marker CD44 to enrich for SCs. This experiment shows that the number of SCs at population confluence is not correlated with the initial plating density of SCs, as measured by both the MFU (Figure 4a) and by the expression of the CD44 (Figure 4b). In this experiment, data was normalized to the MFU and CD44 expression of the cells with an initial plating density of 0% CD44+ cells, i.e., all cells were assumed to be CD44 negative. Starting from this population of CD44 negative cells, a non-negligible proportion of MFU is produced, indicating the presence of a SC population and suggesting, that some CD44-negative cells can later on express CD44. Note, however, that mammospheres formed from CD44-negative populations were unable to passage and generate secondary mammospheres whereas CD44+ mammospheres could be passaged indefinitely. The possibility that the cell sorting methods are not completely efficient cannot be ignored, but cell sorting using the magnetic based autoMACs is quoted as sorting samples with a high degree of accuracy and a sample purity of 99%. For the above-mentioned reasons, we performed an additional experiment in which CD24 and ESA were used in combination with CD44 to further enrich for stem cells (CD44+/24lo/ESA+). Experiments using the combination of cell surface markers corroborated the results observed using CD44. Analysis, 2 days after admixture of cells, showed significant differences (p = 0.04) in the number of mammopsheres produced by populations differing in initial SC densities (Figure 4c). However, after 4 and 7 days of growth, the number of stem cells within the populations had reached equilibrium and no longer correlated with the initial plating density (Figure 4d and 4e). Note that since equilibrium is reached at both days 4 and 7 but cellular confluence is not apparent until day 7, we do not consider confluence to play a role in achieving equilibrium between the populations.

The data were normalized to the MFU of the cells with an initial plating density of 0% CD44+/24lo/ESA+ cells. The formation of a small number of mammospheres (1.1%) in the 0% population suggests the presence of SCs. The CD44+/24lo/ESA+ phenotype is known to enrich for, but not to totally purify SCs [23,24], suggesting further cell surface markers are required to completely isolate the SCs. On the other hand, CD44+/24lo/ESA+ are re-expressed from this population suggesting some CD44-/CD24 hi/ESA- cells can later on express the CD44+/24lo/ESA+ phenotype. The explanation for this could be that the cell sorting methods are not completely efficient. However, using bead calibration this cell sorting method has been shown to have a high degree of accuracy with a 99% purity of sorted cell populations. It may also be possible that CD44 is lost from some stem cells during trypsinisation which would lead to decreased efficiency of cell sorting based on the CD44 cell surface marker.

## Discussion

In this work we showed using a simple mathematical model that reduction of signal intensity in the micro-environment is sufficient for immortalization of a homeostatic normal tissue. By performing *in vitro *experiments in breast carcinoma cells, we provided experimental support for the underlying theory. Despite its simplicity, our model yields extremely powerful tissue homeostasis, returning to almost stationary end cell production even after severe SC depletion (see analytical proof in [4]). This cooperative behavior primarily depends on the sensitivity of individual SCs to neighboring SC density, denoted *QS*.

Assuming that this model is a faithful portrayal of developing tissues, we have asked how such tight control on the integrity of normal tissues can succumb to cancer. Our results suggest that SCs are sensitive to the environmental context. Environmental changes that alter intercellular communication may cause SCs either to fully differentiate and disappear in a relatively short while, or to proliferate perpetually, suggesting that inexhaustible cell proliferation can be induced in the absence of carcinogenic mutations. In such cases mutations leading to tissue invasion and metastasis can be an outcome of extensive SC proliferation, rather than reflecting a replication infidelity of a primordial mutated SC, as suggested elsewhere [14,25] (but see further discussion below).

Based on our results, showing that SC proliferation capacity is attenuated under increased signal intensity and augmented under diminished signal intensity, and on evidence for inflammatory cytokines-mediated tumor growth regulation [26], we hypothesize that chronic inflammation, such as that caused by *H. pylori *in the gastric mucosa, reduces signal intensity in the inflammation site and drives BMDCs to excessively proliferate. Moreover, our simulation results pinpoint the propensity of larger founding SC populations to become immortalized. This implies that significantly more extensive BMDC recruitment contributes to the uncontrollable SC proliferation in chronic, rather than acute, *H. pylori *infection [13]. These hypotheses should be tested experimentally, possibly by tracking the correlations between proliferation and genetic mutations in BMDC residing in inflamed environments.

Our experimental data support the *QS *theory by demonstrating that, as the theory suggests, a fixed proportion of SCs in a cell population reaches steady state conditions, independently of their initial fraction. The experimental evidence was gathered from cell sorting of a breast cancer cell line using the expression of cell surface markers to enrich for SCs and the mammosphere culture technique to assay SC number. Our experimental results suggest that despite different initial fractions of SCs marked by either CD44+ or CD44+/24lo/ESA+, the same steady state is always achieved at, or before, population confluence.

It has been suggested that cell expression of CD44/24 is not fixed and that cancer is organized stochastically, rather than hierarchically, as the SC model suggests. Quintana et al [27] have recently proposed this is the case for melanoma and this has been considered mathematically in [28]. Our experimental results in MCF7 cells, showing that different initial SC proportions yield fixed SC proportions at confluence, cannot be accounted for by the stochastic model, but are readily accounted for by the *QS *model.

A much debated aspect of SC replication is the diverse fate of the two daughter cells that result from each division. In principle, such divisions are considered to be symmetric if the two daughter cells are identical to each other, retaining SC properties, or become committed cells. Divisions are considered to be asymmetric if one daughter cell retains the SC properties of the parent, while the other cell commits to a more differentiated stage [29]. We generalized the different types of SC division into one by assuming independence between the two daughter cell formation. This generalization simplifies our model without compromising its quality, since it allows to examine the forces affecting SC decision to yield either a SC offspring or a DC offspring. A model to evaluate the impact of symmetric and asymmetric SC replication on the expansion of mutant SCs has been recently provided by [30].

The choice between self-renewal and the initiation of differentiation has been suggested to be controlled by specific anatomic structures, termed *stem cell niche *[31]. This niche is mainly composed of the extracellular matrix adhesion molecules, local cell populations and the secreted and cell-surface-bound molecules that these cells generate. In contrast to this theory, the *QS *theory argues that SC fate regulation does not require an externally imposed structure, but, rather, results from spontaneous organization of SCs, and the inter-cell communication in their immediate micro-environment. Nevertheless, it is plausible that micro-environmental maintenance of appropriate signal intensity, e.g., by other selected molecules, or by other factors, such as oxygen tension [32], is enabled by specialized niche structures. Further support for the existence of QS in human tissues should encourage the search for the specific molecules that regulate SC replication via this mechanism. Possible candidates are cytokines or cytokine-like molecules, small molecules which diffuse through gap-junction, or small regulatory RNA molecules that are known to be linked to cancer cell proliferation [5,33,34]. Interestingly, the latter molecules were found to be essential regulators of the *QS *mechanism of several bacteria species [35]. The identification of the *QS *molecules may prove valuable for the manipulation of cancer SCs. These could be therapeutically applied to the tumor micro-environment as decoy SC neighbors, artificially driving cancer SCs to differentiation. This idea underlined our search for the *QS *molecule(s) in breast cancer SCs (BCSCs) [36]. In this work a mathematical model for the major signaling pathways governing mammary BCSC fate-decision was developed. The model suggested that the Wnt pathway inhibitor, Dickhopf1 (Dkk1), was the *QS *molecule in breast cancer, and that high levels of this protein will drive BCSCs into differentiation, leading to tumor elimination. To verify model's predictions, Dkk1's effects on BCSCs were measured, showing that treatment by high Dkk1 concentrations, significantly decreases BCSC counts, both in BC cell line and in cells from primary tumor of a BC patient. We postulated that Dkk1 is a *QS *molecule governing SC fate decision in breast cancer.

Our results further show that replication infidelity, leading to mutations that alter cell kinetic parameters, may also affect the *QS *activity. From Table 1 we learn that shorter life span of DCs, e.g., due to large local death rates, provides a relative advantage to SC proliferation. This implies that drugs that are targeted at DCs, such as those attached to monoclonal antibodies that bind surface molecules expressed on DCs only, will be less effective than anti-proliferative agents. It seems plausible that, in some cases, such drugs may even aggravate cancer SC proliferation, rather than alleviate it. However, appropriately sequenced combinations of these drugs with anti-proliferatives should be tested as well.

## Conclusions

Our work suggests a general mechanism accounting for the regulation of SC replication rate, which integrates in a simple way both environmental and genetic events. *In vitro *experiments in breast carcinoma cell line support the existence of this mechanism. *In vivo *verification and identification of the *QS *molecules will pave the way for a higher level control of SC proliferation in cancer and in tissue engineering.

## Methods

### Mathematical and computational Methods

In our model the set of all graph vertices is the set of all sites having integer coordinates *x*, *y*, *z *in the cubic domain that is determined by the inequalities(2)

*M *being an integer characterizing the size of the cellular matrix, so that the graph has (2·*M *+ 1)^3 ^vertices. Our cellular automata iterative operator, adjusted from the previous model of Agur *et al *[4], is given by

where *K*_1_, *K*_2_, *K*_3_, ... are coefficients representing the magnitude of intercellular communication, that is, the amount of signals reaching a SC from SCs at different proximities; *K*_1_, *K*_2_, *K*_3_, ... are ordered in ascending order according to all possible distances between vertices in the graph; *S*_1_(*v*), *S*_2_(*v*), *S*_3_(*v*), ... denote the corresponding numbers of SCs at those vertices; *L *denotes the SC's sensitivity threshold. In the present work, the threshold value was set to be *L *= 6 unless specified otherwise. We used lower values of *L *on the borders of the cubic domain than inside the domain in order to fit the potential maximal number of neighboring cells. Note that the previous model of Agur *et al *is a special case of the present model, having *K*_1 _= 1, (∀*i *≠ 1) *K*_*i *_= 0, *L *= 6 [4]. The developmental state of a cell is denoted *S*, *D*, or *N*, standing for SCs, DCs and vacant sites, respectively. For simplicity, our model assumes that the DC compartment includes all differentiated descendants of a SC, including progenitor cells, whose limited proliferation is ignored here. The effect of the latter simplification is that each DC in our model represents the cell lineage resulting from one SC differentiation event, so that the numbers of DCs calculated by our model should be, in fact multiplied by a factor of 2^2 ^to 2^10^, depending on the modelled tissue.

The model was implemented in the computer, assuming a cellular matrix whose capacity is ca. 230, 000 cells (assuming *M *= 30 in (2), equaling 61^3 ^vertices); and large simulation experiments were run, each experiment characterized by the initial conditions and kinetic parameter values. The values of Φ, Ψ, Θ were randomly selected from the interval [0; 9], discarding triplets not fulfilling (1). Initial numbers, spatial and age distributions of SCs were randomly selected, where the random choice of initial number of cells was done from the open interval (1; 61^3^) in logarithmical scale.

### Experimental Methods

#### Cell lines

The human breast cancer cell line, MCF7 (ATCC), was maintained in adherent conditions in DMEM (Gibco) with penicillin/streptomycin (100 U/ml penicillin and 100 *μg *streptomycin) (Gibco 15140), L-glutamine (29.2 mg/ml Gibco) and 10% foetal calf serum (100 *μ*l Gibco) in a humidified incubator at 37C at an atmospheric pressure in 5% (v/v) carbon dioxide/air.

#### Cell sorting for CD44+ cells

Following trypsinisation, MCF7 cells were resuspended at ≤ 1 × 10^6 ^in 100 ml buffer (PBS 2 mM EDTA, 0.5% BSA) and incubated for 10 minutes at 40C with fluorescent pre-conjugated primary antibody (CD44-FITC, 1:10 dilution, Beckman Coulter). Cells were washed in PBS and centrifuged at 600 g for 2 minutes. Cells were resuspended in 100 *μl *of buffer with anti-FITC micro-beads (1:10, Miltenyi Biotech) and incubated for 15 minutes at 40C. Cells were washed in PBS, centrifuged at 600 g for 2 minutes and resuspended in 500 *μl *buffer. Cells were sorted using the AutoMACS (Miltenyi Biotech) and the CD44 positive cells were collected.

#### Cell sorting for CD44+/24lo/ESA+ cells

MCF7 cells were resuspended at ≤ 1 × 10^6 ^in 100 ml buffer (PBS 2 mM EDTA, 0.5% BSA) and incubated with fluorescent pre-conjugated primary antibodies (dilution); BEREP4-FITC (1:10, Biomeda), CD44-APC (1:20, BD Pharmingen) and CD24-PE (1:10, Beckman Coulter) for 10 minutes at 40C. The cells were then washed in PBS and centrifuged at 600 g for 2 minutes. Cells were resuspended in 500 ml of 1× Hanks Buffered Saline Solution (HBSS, Invitrogen) after incubation and sorted at 16PSI using the FACS Aria (BD Biosciences).

#### Cell growth assay

After cell sorting for CD44+ or CD44+/24lo/ESA+ cell populations, a single cell suspension was prepared by passing through a syringe and being confirmed by microscopy before plating in adherent conditions. Cells were plated with different proportions of either CD44+ or CD44+/24lo/ESA+ cells admixed with the remaining cell populations. Following magnetic sorting for CD44, positive cells were plated at densities of 0%, 2.5%, 5%, 10%, 32% and 100% and cultured for 7 days. FACS-sorted CD44+/24lo/ESA+ cells were plated at densities of 0%, 1%, 6%, 9% and 12% and cultured for 2, 4 or 7 days. After culture, cells were trypsinised for analysis using flow cytometry and the mammosphere formation assay. Cell sorting experiments were carried out in duplicate. The initial cell population size was 10% relative to confluence.

#### Flow cytometric Analysis of CD44 Expression

Following trypsinisation, MCF7 cells were resuspended at ≤ 1 × 10^6 ^in 100 ml buffer (PBS 2 mM EDTA, 0.5% BSA) and incubated for 10 minutes at 40C with fluorescent pre-conjugated primary antibodies (dilution); CD44-FITC (1:10, Beckman Coulter). Following incubation, cells were washed in PBS and centrifuged at 600 g for 2 minutes. For analysis, cells were resuspended in 500 ml of buffer and fluorescence was measured using the FACS Calibur (BD Bioscience) and analysed using WinMIDI 2.8 software.

#### Mammosphere culture

A single cell suspension was prepared from MCF7 monolayer cultures, which were plated using mixtures of cells sorted for CD44 or CD44/24/ESA as described above, using enzymatic (Trypsin-EDTA) and manual disaggregation (25 gauge needle). The cells were plated at a density of 500 cells/cm^2 ^in non-adherent conditions, in culture flasks coated with (2-hydroxyethylmethacrylate) (poly-HEMA [Sigma]). Cells were incubated for 7 days in mammosphere medium (DMEM: F12 medium supplemented with B27 without vitamin A [diluted 1: 50; Gibco]) and mammary epithelial growth medium aliquot of recombinant human epidermal growth factor (EGF) and gentamicin/amphotericin-B, (SingleQuot) (Lorne Laboratories). Spheres over 50 M were counted after 2, 4, 7 days, for all initial number of SCs. Note that after 7 days the cell population reached confluence, defined as uniform layer cells, roughly occupying 80% of cells on the surface. The percentage of cells plated, which formed spheres, was calculated and is referred to as the percentage mammosphere formation units (%MFU).

#### Statistical Analysis

Data was analyzed using analysis of variance (ANOVA) to determine significant differences between plating different admixed proportions of sorted cells. P values ≤ 0.05 were assumed to be significant.

## Competing interests

The authors declare that they have no competing interests.

## Authors' contributions

ZA contributed the ideas underlying this study, interpreted and revised the Results, and wrote the manuscript. YK contributed the ideas for designing the experiments to examine the model's conclusions, and revised the manuscript. LL simulated the mathematical model and interpreted the results. OUK conceived and wrote the mathematical model and simulation programs, and interpreted the results. HH, RL and RC planned, carried out and described the experiments in the manuscript, RC revised the manuscript.

## Reviewers' comments

*G. Webb*: This paper makes a very strong case for the importance of quorum sensing in the regulation of cancer stem cell proliferation. In vitro experiments showed non-correlation of stem cell fractions at confluence with initial proportions of stem cells, confirming the quorum sensing hypothesis. The results are based on discrete cellular automata models with negative feedback control of cell density on cancer stem cell proliferation, subject to threshold conditions. Can similar results be obtained with continuum partial differential equations models?

*Authors' Response*: In the present work we aimed at modelling a small number of discrete entities (stem cells within a small cancer tissue). We have modelled this system by cellular automata on a regular grid, as we believe this is the most suitable description. Prof. Webb raises an interesting question, namely can similar results be obtained by continuum PDEs? This question should be studied in future research, e.g., when addressing the Quorum Sensing effect in a very large cell population. We are not aware of such continuum approximation of the "ADG model" we used, and wonder whether it will better yield to analysis.

*M. Kimmel*: The Quorum Sensing model considered in the paper is reminiscent of the Stochastic Contact, Voter and Exclusion processes as considered in the well-known monograph by Thomas Liggett. Contact processes framework involves the possibility of transition (with parameter change) from subcriticality to supercriticality, which corresponds to unlimited growth. Although Stochastic Interacting Systems are commonly less sophisticated than the QS model, they may provide insights analogous to those branching process theory provides for population growth models.

*Authors' Response*: Indeed, there exists a similarity between the model we develop and analyze in this work and the models that can be developed and analyzed in the framework of contact processes. Specifically, our model can be represented as stochastic dynamics on finite graph, the vertices being the cells and the edges - the neghbourhood relations. In such a case, the model rules are similar to the classical contact process, in which the state of the vertex is defined (probabilistically) from the states of its negihbours. Note, however, that we have used a discrete time step in stochastic simulations and that the cell in our model can assume more than two possible states and some decisions are only internal state-dependant. It will be very interesting and challenging to find the way to develop and apply the results of the study of contact processes to our Quorum Sensing models, in order to get a better theoretical insight into the model dynamics. We thank the reviewer for making us aware of this option.
